# Loss of Krüppel-like factor-10 facilitates the development of chemical-induced liver cancer in mice

**DOI:** 10.1186/s10020-023-00751-1

**Published:** 2023-11-09

**Authors:** Sung Hwan Yoo, Ji Hae Nahm, Woon Kyu Lee, Hyun Woong Lee, Hye Young Chang, Jung Il Lee

**Affiliations:** 1 Department of Internal Medicine, Gangnam Severance Hospital, Yonsei University College of Medicine, 211 Eonju-Ro, Gangnam-Gu, Seoul, 06273 Republic of Korea; 2 Department of Pathology, Gangnam Severance Hospital, Yonsei University College of Medicine, Seoul, 06273 Republic of Korea; 3 Laboratory of Developmental Genetics, Department of Biomedical Sciences, Inha University College of Medicine, Incheon, 22212 Republic of Korea; 4 Medical Research Center, Gangnam Severance Hospital, Seoul, 06230 Republic of Korea

**Keywords:** Hepatocellular carcinoma, KLF10, Liver fibrosis, TGFβ

## Abstract

**Background:**

Krüppel-like factor 10 (KLF10) is involved in a positive feedback loop that regulates transforming growth factor β (TGFβ) signaling, and TGFβ plays an important role in the pathogenesis of liver disease. Here, we investigated whether *KLF10* deletion affects the development of liver fibrosis and hepatocellular carcinoma (HCC).

**Methods:**

We induced *KLF10* deletion in C57BL/6 mice. Liver fibrosis was induced by feeding a diet high in fat and sucrose (high-fat diet [HFD]), whereas HCC was produced by intraperitoneal administration of N-diethylnitrosamine (DEN). An in vitro experiment was performed to evaluate the role of KLF10 in the cancer microenvironment using Hep3B and LX2 cells. An immunohistochemical study of KLF10 expression was performed using human HCC samples from 60 patients who had undergone liver resection.

**Results:**

*KLF10* deletion resulted in an increased DEN-induced HCC burden with significant upregulation of *SMAD2*, although loss of KLF10 did not alter HFD-induced liver fibrosis. DEN-treated mice with *KLF10* deletion exhibited increased levels of mesenchymal markers (*N-cadherin* and *SNAI2*) and tumor metastasis markers (*matrix metalloproteinases 2* and *9*)*. KLF10* depletion in Hep3B and LX2 cells using siRNA was associated with increased invasiveness. Compared with co-culture of *KLF10*-preserved Hep3B cells and *KLF10*-intact LX2 cells, co-culture of *KLF10*-preserved Hep3B cells and *KLF10*-depleted LX2 cells resulted in significantly enhanced invasion. Low KLF10 expression in resected human HCC specimens was associated with poor survival.

**Conclusion:**

The results of this study suggest that loss of KLF10 facilitates liver cancer development with alteration in TGFβ signaling.

## Introduction

Chronic liver injury caused by infectious, inflammatory, and metabolic disorders may result in liver fibrosis, the strongest risk factor for hepatocellular carcinoma (HCC) (Alter and Seeff 2000; Caldwell et al. 2004). Although nearly 90% of HCCs develop in cirrhotic livers (Llovet et al. 2021), fewer than 5% of patients with cirrhosis undergo progression to HCC annually (West et al. 2017). Despite recent advances in the understanding of the molecular pathogenesis of HCC and the identification of driver mutations, the key mechanisms involved in HCC development from cirrhosis are unclear.

Transforming growth factor β (TGFβ) plays an important role in the development of liver fibrosis and HCC by altering the composition of the microenvironment (Fabregat et al. 2016; Lee et al. 2016). TGFβ plays key roles in the plasticity of hepatic stellate cells and macrophages, which are the two major cells involved in liver fibrosis (Dewidar et al. 2019). TGFβ plays a dual role in HCC development; it suppresses tumor development in early stages and promotes tumor progression in later stages (Caja et al. 2007; Russell et al. 1988; Valdes et al. 2004; Wilkes et al. 2005). The mechanisms involved in shifting the role of TGFβ from tumor suppression to the regulation of tumor progression require further investigation.

The Krüppel-like factor (KLF) family shares a three-C_2_H_2_ zinc finger DNA-binding domain with a Krüppel linker between the zinc fingers (VHS Chang et al. 2012). One KLF family member, the human homologue of KLF10 (also known as the TGFβ-inducible early gene 1 [TIEG1]), is the product of a TGFβ-inducible early-response gene in osteoblastic cells (Subramaniam et al. 1995). KLF10 is involved in a positive feedback loop that regulates TGFβ signaling by inducing *SMAD2* expression and inhibiting the expression of the inhibitory *SMAD7* gene (Johnsen et al. 2002a; Johnsen et al. 2002b). Additionally, an increased intracellular level of KLF10 facilitates the anti-proliferative and pro-apoptotic effects of TGFβ on epithelial cell growth (Ellenrieder 2008). With respect to liver fibrosis, we previously reported that high-fat diet (HFD)-induced liver fibrosis was accompanied by increased KLF10 expression (Kim et al. 2014a). In the present study, we investigated whether KLF10 plays an important role in generation of liver fibrosis using *KLF10*-deleted transgenic mice and examined the impact of *KLF10* deletion in HCC development.

## Materials and methods

### Animal studies

*KLF10* knockout (KO) mice were provided by Prof. Woon-Kyu Lee (Inha University College of Medicine, Incheon, Republic of Korea). The mice developed, grew, and reproduced normally, as previously described (Song et al. 2012; Subramaniam et al. 2005). *KLF10* KO mice were compared with wild-type (WT) C57BL/6 J mice. Each experimental group included ≥ 5 animals. Randomization was not used to allocate the animals to the control and treatment groups. All experimental animals that were properly sacrificed were included in the analysis. Investigators who performed the histologic analysis were blinded to the animals’ group allocations.

The animal experimental procedures and protocols were approved by the Institutional Animal Care and Use Committee of Gangnam Severance Hospital, Yonsei University College of Medicine (permit nos.: 2013–0173 and 2015–0049). The study was performed in accordance with the guidelines of the Institutional Animal Care and Use Committee.

### Liver fibrosis induction by HFD

Fatty liver-associated liver fibrosis was induced by feeding a diet high in saturated fats, cholesterol, and sucrose (i.e., HFD) as previously reported (JK Kim et al., 2014b). Male *KLF10* KO and WT mice were fed an HFD (#5053*; PicoLab, Bethlehem, PA, USA) consisting of 15% anhydrous milkfat, 1.0% cholesterol, and 50% sucrose. Either a standard diet (SD) or HFD was administered to 6-week-old mice for 24 weeks before sacrifice. All sacrificed animals were included in the analysis.

### HCC induction

HCC was chemically induced using N-diethylnitrosamine (DEN). DEN produces reactive ethyl diazonium ions in the liver, altering the expression levels of tumor promoting and/or suppressing genes (Swenberg et al. 1991); this leads to HCC development without the onset of liver fibrosis (F Heindryckx et al., 2010; M Kushida et al. 2011). In DEN-induced mouse hepatocarcinogenesis, altered hepatocellular foci, such as glycogen-rich clear cells, have been used as markers of preneoplastic lesions (Kushida et al. 2011). In the present study, DEN was intraperitoneally administered once weekly to 2-week-old male *KLF10* KO and WT mice for 8 weeks. DEN was administered at a dose of 20 mg/kg bodyweight for 2 weeks, followed by 50 mg/kg bodyweight beginning at the age of 4 weeks; this treatment was continued for 6 weeks. Mice were sacrificed at the age of 24 weeks. Untreated age-matched WT mice were sacrificed and served as controls. All sacrificed animals were included in the analysis.

### Histologic evaluation of animal liver samples

The liver samples from sacrificed animals were macroscopically evaluated before storage. HCC and non-HCC tissues were separately collected and snap-frozen in liquid nitrogen. Hematoxylin and eosin staining was performed to evaluate morphological changes. Sirius red staining was used to evaluate fibrotic changes in the liver. Fibrosis was semi-quantitatively evaluated by expressing the fibrosis ratio using an image analysis system as described in a previous study with modification (O'Brien et al. 2000). The total area was the sum of the area of the microscopic fields, including the parenchyma and fibrosis. For each slide, the area of fibrosis was evaluated in 20 consecutive fields at a magnification of 40 × and averaged.

### Patients, tissues, immunohistochemistry

We retrospectively reviewed 277 patients with HCC who had undergone curative liver resection between January 2006 and December 2016 at Gangnam Severance Hospital, Yonsei University College of Medicine, Seoul, Korea. Paraffin-embedded HCC samples of 60 patients were subjected to histopathological analysis. This study protocol was approved by the Institutional Review Board of Gangnam Severance Hospital, Yonsei University College of Medicine (permit no.: 3-2015-0177). The need for informed consent was waived by the Institutional Review Board because the researchers only accessed the database for analysis purposes and all personal information was blinded by coding. Immunohistochemical staining was performed using mouse monoclonal antibody against KLF10 (sc-130408; Santa Cruz Biotechnology, Santa Cruz, CA, USA; 1:500 dilution) (Chang et al. 2012; Peng et al. 2019). Immunopositivity was assessed with respect to cellular localization, intensity, and distribution as previously described (Hsu et al. 2014; Pandya et al. 2004). KLF10 expression was quantified using a visual grading system based on the extent of staining (E) (percentage of tumor cells: 0, none; 1, 1–30%; 2, 31–60%; 3, > 60%) and intensity of staining (I) (0, none; 1, weak staining; 2, moderate staining; 3, strong staining). The E and I values were multiplied (E × I) to calculate the EI score (0–9). EI scores of < 3 and ≥ 3 indicated low and high expression levels, respectively.

### Cell culture, transfection, and conditioned medium

The human HCC cell line Hep3B (KCLB #88064) (RRID: CVCL_0326) and human hepatic stellate cell line LX2 (cat #SCC064) (RRID: CVCL_5790) were purchased from Korean Cell Line Bank (Seoul, Republic of Korea). The human cell lines had been authenticated using short tandem repeat profiling within 3 years prior to this study. All experiments were performed using mycoplasma-free cells (EMD Millipore, Temecula, CA, USA), as previously described (Friedman et al. 1992; Hiron et al. 1992). Transient depletion assays were performed using DharmaFECT 1 (Dharmacon, Lafayette, CO, USA), in accordance with the manufacturer’s recommendations. SmartPool siRNA against *KLF10* (L-006566-00) and control siRNA (D-001206-13) were purchased from Dharmacon. Briefly, 25 nM siRNA ON-TARGETplus SmartPool (L-006566-00) was transfected with a plating density of 3 × 10^5^ cells per well in a six-well plate.

### Transwell invasion assay and co-culture

The invasive abilities of Hep3B and LX2 cells were evaluated in vitro using a Transwell chamber system with 8.0-μm pore polycarbonate filter inserts (Corning Costar Corp., Cambridge, MA, USA), as previously described (Prenzel et al. 2011). The lower side of the filter was coated with 10 μL of gelatin (1 mg/mL), whereas the upper side was coated with 10 μL of Matrigel. The effect of KLF10 on cell invasion was investigated at 24 h after siRNA transfection by seeding 3 × 10^5^ cells in the upper part of the filter. After 24 h of incubation at 37 °C with 5% CO_2_, the upper surface of the membrane was scrubbed using a cotton swab; cells on the lower surface of the membrane were fixed with 4% paraformaldehyde, then stained with crystal violet. The numbers of cells that migrated through the Matrigel were counted in five random fields under a microscope at 200 × magnification.

For co-culture of Hep3B and LX2 cells, cells were cultured using hanging cell culture inserts (pore size, 1 µm; Falcon) to separate the cell populations. Wells and inserts with media were stabilized for 24 h at 37 °C, in accordance with the manufacturer’s recommendations. Hep3B cells were seeded in the insert (3 × 10^3^ cells/cm^2^) and incubated overnight in Dulbecco’s modified Eagle medium with 10% fetal bovine serum. LX2 cells (3 × 10^5^ cells/cm^2^) were seeded on the upper part of the filter of the Transwell chamber system, then stabilized for 24 h. On the following day, LX2 cells were subjected to siRNA transfection (*KLF10* or control) using DharmaFECT 1 (Dharmacon), in accordance with the manufacturer’s recommendations. The plate with LX2 cells was placed below the culture insert with Hep3B cells. The culture was continued for an additional 24 h, and the invasion assay was performed as previously described.

### RNA isolation and real-time polymerase chain reaction (PCR)

Total RNA was extracted from frozen whole liver (in the HFD experiment), from the tumor sites of the liver (in the DEN experiment), or from isolated cells using TRIzol reagent (Invitrogen, Carlsbad, CA, USA) and Qiagen mini columns (Qiagen Inc., Valencia, CA, USA) in accordance with the manufacturer’s recommendations. RNA samples were quantified via spectrophotometry. RNA integrity was assessed by agarose gel electrophoresis and ethidium bromide staining. The RNA samples were diluted in RNase-free water and stored at − 70 °C until use. In total, 5 μg of RNA were reverse-transcribed via RNA PCR (version 1.2; TaKaRa Bio Inc., Tokyo, Japan) in accordance with the manufacturer’s recommendations. Oligonucleotide primers and TaqMan probes for *TGFβ*, *SMAD2*, *SMAD3*, *SMAD7*, *collagen α1(I)* [*Col1α(I)*], *α smooth muscle actin (SMA)*, *lecithin retinol acyltransferase (Lrat)*, *E-cadherin*, *N-cadherin*, *SNAI2*, and *matrix metalloproteinases (MMPs) 2* and *9* were used; *18S* was used an internal control. Probes were obtained from Applied Biosystems (Perkin-Elmer/PE Applied Biosystems, Foster City, CA, USA) and prepared in ready-to-use format with Assays-on-Demand Gene Expression Products. TaqMan probes were labeled at the 5’ end with the reporter dye FAM and at the 3’ end with the minor groove binder nonfluorescent quencher. Quantitative PCR was performed in triplicate for each sample using the Step One Plus Real Time System (Applied Biosystems). Each 20-μL reaction was performed using 10 μL of TaqMan Fast Universal Master Mix (Applied Biosystems, Darmstadt, Germany), 1 μL of Gene Expression Mix, and 2 μL of cDNA diluted in 7 μL of RNase-free water. The thermocycler protocol was 20 s at 95 °C, followed by 40 cycles of 5 s at 95 °C and 20 s at 60 °C. The fold-change in mRNA expression levels of target genes relative to the endogenous 18S control was calculated as previously described (KJ Livak and TD Schmittgen, 2001).

### Statistical analysis

All results are presented as means ± standard errors of the mean. Data were analyzed using nonparametric tests (Kruskal–Wallis or Mann–Whitney) or one-way analysis of variance with Tukey’s post hoc test. For patients, overall survival was analyzed according to KLF10 expression using the Kaplan–Meier method. Differences in survival rate were compared using the log-rank test. *P*-values < 0.05 were considered statistically significant. Statistical analysis was performed using SPSS software (version 23; IBM Corp., Armonk, NY, USA).

## Results

### Effect of *KLF10* deletion on TGFβ signaling in the untreated liver

**Fig. 1:**
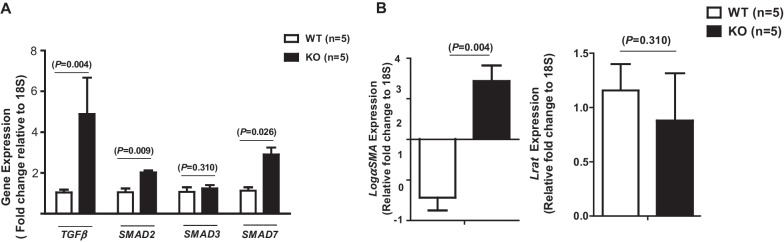
Gene expression in the untreated liver of *KLF10* wild-type (WT) and knockout (KO) mice. **A** The expression of TGFβ-related signaling genes was evaluated by real-time polymerase chain reaction in WT (n = 5) and *KLF10* KO (n = 5) mice liver with no treatment. **B** The expression of hepatic stellate cell activation-related genes was compared between WT (n = 5) and *KLF10* KO (n = 5) mice liver with no treatment

KLF10 is a critical effector of the TGFβ/SMAD signaling pathway (Johnsen et al. 2002b). Male WT mice (n = 5) and *KLF10* KO mice (n = 5) fed an SD were sacrificed at the age of 8 weeks to evaluate the effect of *KLF10* deletion on gene expression. In *KLF10* KO mice, the liver exhibited increased expression levels of *TGFβ*, *SMAD2*, and the inhibitory factor *SMAD7*, although the expression of *SMAD3* was unaffected (Fig. 1A). αSMA is upregulated in activated hematopoietic stem cells (HSCs) and is closely associated with liver fibrosis (N Roehlen et al., 2020). In untreated *KLF10-*deleted mice, the liver exhibited significantly upregulated expression of *αSMA*; the expression of *Lrat*, which is expressed in quiescent HSCs, was unaffected (Fig. 1B).

### *KLF10* deletion did not alter HFD-induced liver fibrosis

**Fig. 2:**
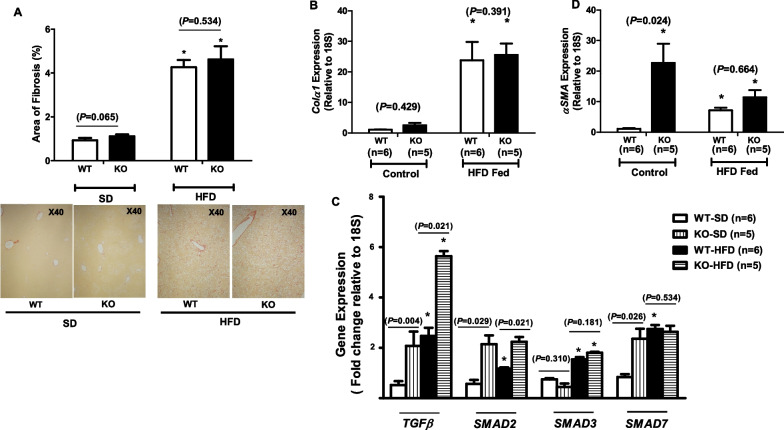
Liver fibrosis induction and gene expression in high-fat diet (HFD)-treated liver of *KLF10* wild-type (WT) and knockout (KO) mice. **A** Liver tissue was stained for collagen deposition using Sirius red staining, and a morphometric analysis was performed. **B** The expression of *Colα1* was evaluated by real-time polymerase chain reaction in WT mice fed a standard diet (SD) (n = 6), *KLF10* KO mice fed an SD (n = 5), WT mice fed an HFD (n = 6), and *KLF10* KO mice fed an HFD (n = 5). **C** Expression of TGFβ-related signaling genes. **D** Expression of *αSMA*. *P < 0.005 compared with SD-fed WT or KO mice

Liver fibrosis and cirrhosis are strong predisposing factors for HCC (EAS Liver 2018; Marrero et al. 2018). Additionally, TGFβ facilitates liver fibrosis. Studies have shown that TGFβ is important for the activation and proliferation of HSCs, which are the main cellular components of liver fibrosis (Greuter and Shah 2016). Additionally, we have shown that HFD-induced liver fibrosis is accompanied by increased KLF10 expression (JK Kim et al., 2014a). Therefore, we investigated how *KLF10* deletion affected TGFβ signaling and liver fibrosis in mice with HFD-induced chronic liver inflammation. Male *KLF10* WT (n = 6) and KO (n = 5) mice were fed an HFD, high in saturated fats, cholesterol, and sucrose, for 24 weeks; male WT mice (n = 6) and KO (n = 5) were fed an SD for 24 weeks and served as controls. The HFD increased liver fibrosis, as evaluated by Sirius red staining (Fig. 2A), as well as the mRNA expression level of *Col1α* in the livers of *KLF10* WT and KO mice, without significant differences between groups (Fig. 2B). In both WT and KO mice, the provision of an HFD induced increases in the expression levels of *TGFβ* and downstream signals *SMAD3* compared with the SD-fed controls (Fig. 2C). Although the HFD-fed WT mice showed increased *SMAD2* and *SMAD7* expression, the HFD-fed KO mice did not show significant increases compared with the SD-fed KO controls. *TGFβ*, *SMAD2*, and *SMAD7* expression levels were significantly higher in *KLF10* KO control mice than in WT mice, whereas *SMAD3* expression levels showed no significant differences. *TGFβ* and *SMAD2* expression were significantly higher in HFD-fed *KLF10* KO than in HFD-fed WT mice. Overexpression of *αSMA* was observed in the livers of HFD-treated WT and *KLF10* KO mice, without significant differences between the groups (Fig. 2D).

### *KLF10* deletion increased the liver cancer burden in DEN-treated mice

**Fig. 3:**
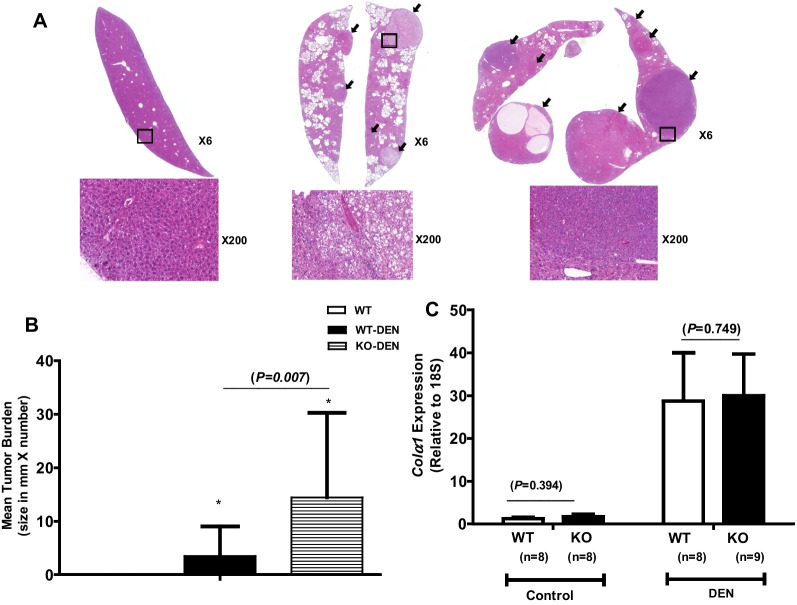
Induction of hepatocellular carcinoma (HCC) by diethylnitrosamine (DEN) treatment in *KLF10* wild-type (WT) and knockout (KO) mice. **A** Two-week-old *KLF10* KO (n = 9) and WT (n = 8) mice received intraperitoneal DEN injections weekly for 8 weeks. DEN-injected mice were sacrificed at the age of 24 weeks. Liver specimens were stained with hematoxylin and eosin (H&E) and evaluated for HCC development. Representative images of HCC (arrow) are shown. The boxed part of HCC in the low-magnification image (6 ×) is magnified (200 ×) and presented. **B** The HCC burden (size in mm × tumor number) was evaluated under microscopy after H&E staining in *KLF10* WT (n = 8) and KO (n = 9) mice. (**C** Assessment of liver fibrosis by *Colα1* mRNA expression after DEN treatment in *KLF10* WT (n = 8) and KO (n = 9) mice within liver tumor sites. Age-matched WT (n = 8) or KO (n = 8) mice with no treatment served as the controls. *P < 0.005 compared with SD-fed WT or KO mice

**Fig. 4:**
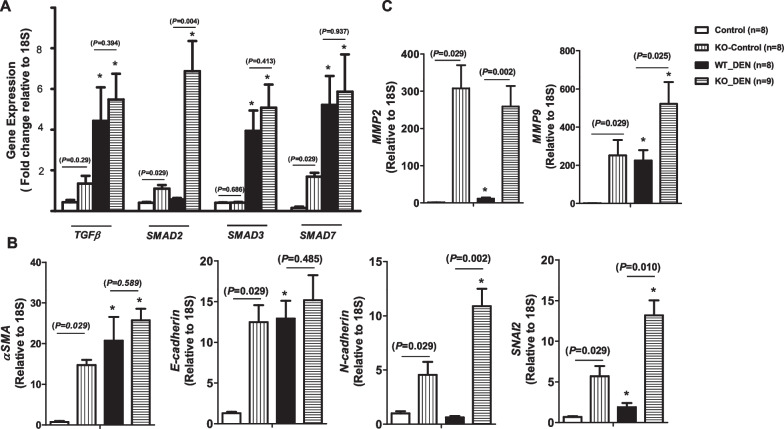
Gene expression in tumor sites of diethylnitrosamine (DEN)-treated liver of *KLF10* wild-type (WT) and knockout (KO) liver. **A** The expression of TGFβ-related signaling genes was measured in WT mice without treatment (n = 8), KO mice without treatment (n = 8), WT mice with DEN treatment (n = 8), and KO mice with DEN treatment (n = 9). **B** The expression of epithelial-to-mesenchymal transition–related markers was evaluated. **C** The expression of genes related to tumor invasiveness was assessed. *P < 0.005 compared with SD-fed WT or KO mice

In the present study, 2-week-old male *KLF10* KO (n = 9) and WT (n = 8) mice received weekly intraperitoneal injections of DEN for 6 weeks. DEN-injected mice were sacrificed at the age of 24 weeks. Age-matched male WT mice without any treatment (n = 8) served as controls. Liver specimens were stained with hematoxylin and eosin, then evaluated for HCC development (Fig. 3A). HCC nodules were counted and measured via microscopy at a low magnification. The HCC burden was defined as the longest diameter of the tumor (mm) × tumor number for each liver specimen. When HCC was induced by DEN treatment, *KLF10* deletion resulted in a significantly greater tumor burden compared with the burden in DEN-treated WT mice (Fig. 3B). The extent of liver fibrosis did not significantly differ between groups (Fig. 3C). DEN treatment was associated with increased *TGFβ*, *SMAD3*, and *SMAD7* expression in both DEN-treated WT and KO mice compared with their untreated counter parts (Fig. 4A). However, there were no significant differences in the extent of the increment between DEN-treated WT and KO mice. *SMAD2* expression was significantly upregulated by *KLF10* deletion, and treatment with DEN induced a further increase in *SMAD2* expression (Fig. 4A).

### *KLF10* deletion upregulated markers of mesenchymal cells in the liver

To explore how *KLF10* deletion promotes HCC development in DEN-treated mice, we evaluated the expression levels of *αSMA, E-cadherin, N-cadherin,* and *SNAI2* (i.e., markers of epithelial-to-mesenchymal transition [EMT]). DEN-treated WT mice exhibited increased expression levels of *αSMA*, *E-cadherin,* and *SNAI2* (Fig. 4B). *KLF10* deletion resulted in increased *αSMA*, *E-cadherin, N-cadherin,* and *SNAI2*. Although DEN treatment in *KLF10* KO mice further increased *αSMA*, *N-cadherin,* and *SNAI2* expression, the level of *E-cadherin* did not show significant alteration after DEN treatment (Fig. 4B).

### *KLF10* deletion enhanced HCC invasiveness genes

MMPs facilitate EMT by increasing invasion and metastasis (BN Smith and NA Bhowmick, 2016). Although not typically present in liver cells, MMP2 is expressed in HCC; in this context, it enhances tumor invasion and metastasis (Chen et al. 2017; Chen et al. 2012). MMP9 and MMP2 are poor prognostic factors in HCC. *KLF10* deletion resulted in enhanced *MMP2* and *MMP9* gene expression in untreated KO mice, and DEN treatment further increased *MMP9* expression in *KLF10* KO mice (Fig. 4C). In *KLF10* WT mice, DEN treatment was associated with increased *MMP2* and *MMP9* expression (Fig. 4C). However, because untreated *KLF10* KO mice already had increased *MMP2* and *MMP9* levels, the levels of both *MMP2* and *MMP9* in DEN-treated WT mice remained lower than those in DEN-treated KO mice.

### *KLF10*-depleted LX2 cells promote invasiveness in Hep3B cells

**Fig. 5:**
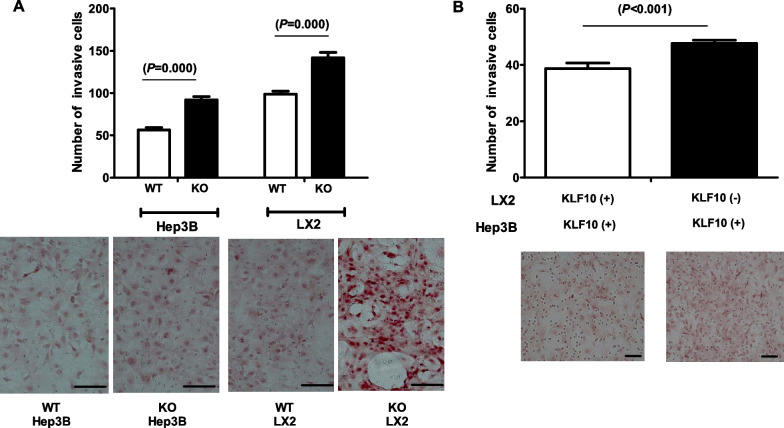
Invasion of Hep3B and LX2 cells. **A** Invasion of Hep3B and LX2 cells was determined using the Transwell assay. Transient *KLF10* deletion was induced using siRNA. The number of invasive cells was counted in five random fields under a microscope at 200 × magnification and is shown as the mean ± standard deviation. Representative images from the experiment are shown. **B** Invasion of Hep3B cells was assessed after co-culture with LX2 cells with *KLF10* either deleted or preserved. Hep3B and LX2 cells were cultured using hanging cell culture inserts (1-µm pore size, Falcon) to separate cell populations. Hep3B cells were seeded in the insert (3 × 10^3^ cells/cm^2^) and allowed to attach overnight in Dulbecco’s modified Eagle medium with 10% fetal bovine serum. LX2 cells (3 × 10^5^ cells/cm^2^) were seeded on the upper part of the filter of the Transwell chamber system, and siRNA transfection (*KLF10* or control) was performed. The plate with LX2 cells was placed below the culture insert with Hep3B cells. The number of invasive cells was counted in five fields under a microscope at 200 × magnification and is shown as the mean ± standard deviation. Representative images from the experiment are shown. Scale bar = 50 µm

The effect of *KLF10* depletion on the invasiveness of Hep3B and LX2 cells was evaluated using siRNA. *KLF10* depletion promoted invasion in Hep3B and LX2 cells (Fig. 5A). Activated HSCs are important cellular components in the tumor microenvironment (Coulouarn and Clement 2014; Friedman 2008). A previous study revealed that when co-cultured with HSCs, HCC cells exhibited significant increases in proliferation and migration (Amann et al. 2009). To determine whether *KLF10* depletion alters the effects of HSCs on Hep3B, Hep3B cells were co-cultured with *KLF10*-preserved or *KLF10*-depleted HSCs. The invasiveness of Hep3B cells was significantly enhanced during co-culture with *KLF10*-depleted LX2 cells (Fig. 5B).

### Low KLF10 expression in human HCC is associated with poor survival

**Fig. 6:**
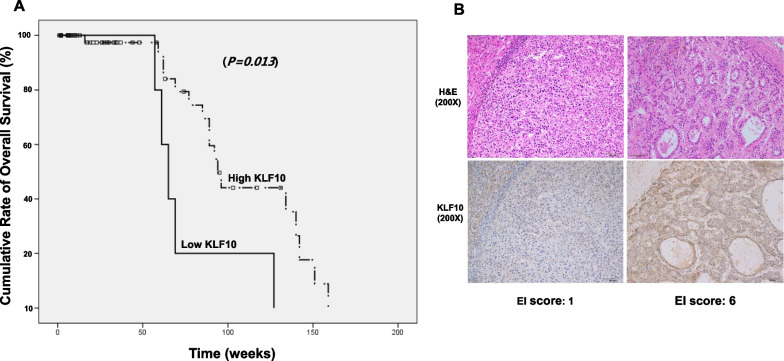
Survival of patients after curative HCC resection according to KLF10 expression in the resected tumor after immunohistochemical staining. **A** The combination of the extent (E) and intensity (I) of staining was obtained by calculating E × I to give the EI score, which ranged from 0 to 9. EI scores < 3 indicated low expression, and EI scores ≥ 3 indicated high expression. **B** Representative immunohistochemistry of KLF10 in human HCC tissue of high and low EI score immunostaining are shown

Patients who underwent liver resection for treatment of HCC from January 2006 to December 2016 were retrospectively evaluated. Of 277 patients, 60 patients with HCC who underwent curative liver resection with a follow-up period of > 12 months were selected, and HCC samples were subjected to histopathological analysis. These 60 patients’ demographic findings are presented in Table 1. The EI score ranged from 0 to 9, with EI scores of < 3 and ≥ 3 indicating low and high expression levels, respectively. Compared with a high EI score, a low EI score was associated with a significantly lower survival rate (Fig. 6A). While immunohistochemistry revealed strong KLF10 expression in the non-tumor hepatocytes, the tumor site showed various intensities and extents of KLF10 staining. Representative images of KLF10-positive tumor cells are shown in Fig. 6B.

**Table 1:** Baseline characteristics of patients that underwent liver resection due to HCC

Variables	n = 60
Age, years, median (range)	51 (30–81)
Gender (M:F)	49:11
Etiology of liver disease	
HBV	43 (71.7)
HCV	4 (6.7)
Others	13 (21.7)
Serum AFP, ng/mL (%)	
< 200	53 (88.3)
≥ 200	7 (11.7)
Tumor number (%)	
Single	52 (86.7)
Multiple	8 (13.3)
Tumor size	
≤ 5 cm	52 (86.7)
> 5 cm	8 (13.3)
Existence of microvascular invasion (%)	16 (26.7)
Edmonson grade (%)	
Grade 1, 2	25 (41.7)
Grade 3, 4	35 (58.3)
Existence of Pathologic Cirrhosis (%)	48 (80.0)
KLF10 Expression	
High	54 (90.0)
Low	6 (10.0)

*AFP* alpha-fetoprotein; *KLF10* Kruppel-like factor 10

## Discussion

This study showed that *KLF10* deletion resulted in an increased incidence of DEN-induced HCC in mice, without the onset of liver fibrosis. Loss of KLF10 led to enhanced migration of malignant hepatocytes and hepatic stellate cells.

*KLF10*, a TGFβ early responsive gene, is involved in a positive feedback loop with respect to TGFβ signaling. Therefore, we hypothesized that modulation of *KLF10* expression would alter the extent of liver fibrosis in a HFD-induced fatty liver mice model. Unexpectedly, HFD challenge did not lead to worsening of liver fibrosis in *KLF10* KO mice compared with WT mice. Although the livers from both HFD-treated WT and *KLF10* KO mice showed increased expression of *TGFβ* and *SMAD3* compared with those of the respective controls*, TGFβ* of HFD-treated *KLF10* KO mice was further increased compared with that of HFD-treated WT mice. The increase in *SMAD3* in WT and KO mice after DEN treatment showed no significant differences between the two groups. *SMAD2* and *SMAD3* have both overlapping and distinct roles in the TGFβ signaling pathway; a previous study showed that *SMAD3* is important for collagen production by HSCs (Zhang et al. 2015). The lack of a significant difference in *SMAD3* expression after DEN treatment in WT and KO mice may partially explain the absence of changes in liver fibrosis after *KLF10* deletion. Because KLF10 is reportedly involved in a positive feedback loop of TGFβ signaling, increased TGFβ expression in the liver of *KLF10* KO mice was not expected. However, cellular responses involving both TGFβ and KLF10 are dependent on the cell type (Itoh and ten Dijke 2007), and TGFβ expression was significantly increased after hepatectomy in *KLF10* KO mice (Heo et al. 2017). Thus, a further study with a different fibrosis model is necessary.

Although our results did not reveal a significant effect of *KLF10* deletion on liver fibrogenesis, the loss of KLF10 led to increased incidence of DEN-induced HCC in mouse liver, suggesting that KLF10 has a tumor suppressor role. Our animal experimental results are in accordance with the result of KLF10 expression in the human HCC specimens, showing decreased survival with decreased KLF10 expression after HCC resection. Additionally, our study provides evidence that KLF10 has a suppressive role during HCC development and progression. The loss of KLF10 led to the upregulation of genes associated with EMT and tumor metastasis. EMT is a transdifferentiation process with a central roles in cancer metastasis and the development of stem cell-like features (Valastyan and Weinberg 2011). TGFβ is among the most potent inducers of EMT; in vitro analyses have shown that stimulation of primary hepatocytes with TGFβ can induce EMT (Caja et al. 2011; Dooley et al. 2008). The loss of KLF10 led to significant upregulation of *TGFβ* and other mesenchymal markers, such as *SNAI2* and *N-cadherin,* in mouse liver after DEN treatment. MMPs have key roles in promoting the invasive and metastatic abilities of malignant tumor cells. MMP2 is not typically present in liver cells but is expressed in HCC cells (Wang et al. 2014). Similar to MMP2, MMP9 plays a major role in tumor angiogenesis. MMP9 overexpression in HCC leads to a higher TNM stage and a poor prognosis (Chen et al. 2012). The present study revealed significantly higher expression levels of *MMP2* and *MMP9* after *KLF10* deletion in the liver of DEN-treated mice, compared with the liver of *KLF10*-preserved WT mice. In addition to the in vivo findings, our in vitro experiment revealed increased migration of Hep3B cells after siRNA-mediated depletion of *KLF10*.

Activated HSCs promote the proliferation and migration of liver cancer cells, both in vivo and in vitro (Amann et al. 2009). Our study revealed increased migration of Hep3B cells co-cultured with *KLF10-*depleted LX2 cells compared with the migration of Hep3B cells co-cultured with *KLF10-*intact LX2 cells. These findings suggest that *KLF10* deletion facilitates the migration of liver cancer cells and enhances the tumorigenic effect of the cancer microenvironment. However, the results of our study regarding the effect of KLF10 loss on liver fibrosis and HCC development should be validated by different fibrosis and HCC models because some etiologies of chronic liver disease are more likely to induce liver fibrosis and HCC in clinical settings (Giannelli et al. 2014).

## Conclusion

The results of this study suggest that loss of KLF10 facilitates liver cancer development with alteration in TGFβ signaling. Loss of KLF10 led to the upregulation of the mesenchymal cell markers *N-cadherin* and *SNAI2* and the invasiveness markers *MMP2* and *MMP9. KLF10* inhibition enhanced the metastatic function of liver cancer cells and the tumor-enabling function of HSCs. In addition, *KLF10*-depleted liver cancer cells and HSCs demonstrated increased invasiveness. These results support the idea that KLF10 is involved in the tumor-suppressing role of TGFβ and that loss of KLF10 promotes cancer development and progression. However, this study involved only a single HCC animal model, and a relatively small number of human samples with a limited follow-up period. Further studies using different HCC models and other cohorts of patients with HCC are necessary to validate our results and investigate the role of KLF10 in HCC.

## Data Availability

The data that support the findings of this study are available from the corresponding author upon reasonable request.

## Abbreviations

αSMA: Alpha smooth muscle actin
DEN: Diethylnitrosamine
EMT: Epithelial-to-mesenchymal transition
HCC: Hepatocellular carcinoma
HFD: High-fat diet
KO: Knockout
Lrat: Lecithin retinol acyltransferase
MMP: Matrix metalloproteinase
SD: Standard diet
TGF: Transforming growth factor
TIEG: Transforming growth factor β inducible early gene
WT: Wild type
